# Fungal Infections of the Ear in Immunocompromised Host: a Review

**DOI:** 10.4084/MJHID.2011.003

**Published:** 2011-01-14

**Authors:** Borlingegowda Viswanatha, Khaja Naseeruddin

**Affiliations:** 1 Professor of ENT, Victoria Hospital, Bangalore Medical College & Research Institute, Bangalore. INDIA; 2 Professor of ENT, Joint Director of Medical Education, Bangalore. INDIA

## Abstract

Otomycosis is a fungal infection of the external ear; middle ear and open mastoid cavity.^1^ Meyer first described the fungal infection of the external ear canal in 1884. External ear canal has an ideal warm humid environment for the proliferation of fungus.^2^ Although this disease is rarely life threatening, it can presents a challenging and frustrating situation for the otologist and patients due to long term treatment and high rate of recurrence.^3^ Otomycosis is seen more frequently in immunocompromised patients as compared to immunocompetent persons. Recurrence rate is high in immunocompromised patients and they need longer duration treatment and complications are more frequent in immunocompromised patients.

In the recent years; opportunistic fungal infections are gaining greater importance in human medicine as a result of possibly huge number of immunocompromised patients.^4^ In immunocompromised patients, it is important that the treatment of otomycosis be vigorous, to minimize complications such as hearing loss, tympanic membrane perforations and invasive temporal bone infection.^5^ Fungal cultures are essential to confirm the diagnosis.

Hematological investigations play a very important role in confirming the diagnosis and immunity status of the patients. In diabetic patients with otomycosis, along with antifungal therapy, blood sugar levels should be controlled with medical therapy to prevent complications.

## Introduction

Otomycosis or fungal otitis externa has typically been described as fungal infection of the external auditory canal with infrequent complications involving the middle ear.^3^ Fungi causes 10% of all cases of otitis externa.^6^ In the recent years there has been an increase in the incidence as a result of possibly huge number of immunocompromised patients. In the past, there were controversies regarding the prevalence and even existence of otomycosis. It is now considered to be a definitive clinical entity and a continuing problem.^1^ Although there has been controversy with respect to whether the fungi are the true infective agents versus mere colonization of the species as a result of compromised local host immunity secondary to bacterial infection, most clinical and laboratory evidence shows that otomycosis as a true pathological entity.^3^

General cellular immunity is reduced in situations such as diabetes, steroid administration, HIV infection, chemothraphy and malignancy (especially those involving cells of immune system).This makes an immunocompromised host susceptible to fungal infections. Normal bacterial flora is one of the host defense mechanism against fungal infections. This mechanism is altered in patient patients using antibiotics ear drops and cause otomycosis.^2^

Otomycosis is sporadic and caused by a wide variety of fungi, most of which are saprobe occurring in diverse type of environmental material.^4^ It affects 10% of the population in their life time.^6^ Fungi are abundant in soil or sand which contains decomposing vegetable matter. This is desiccated rapidly in tropical sun and blown in wind as small dust particles. The air borne fungal spores are carried by water vapors, a fact which correlates the higher rates of infection, monsoon when relative humidity rises to 80%.^7^ Many studies have shown that the incidence was more common in third decade of life. Higher incidence in young adults may be attributed to the fact that these people are more exposed to the mycelia, whereas extreme age groups are not exposed to the pathogens.^3^,^8^,^9^,^10^

Common symptoms of otomycosis are itching, ear pain, ear discharge, blocking decreased hearing and tinnitus.^3^,^8^,^10^ The correct diagnosis of otomycosis requires a high index of suspicion, given that the most common presenting symptoms, otalgia and otorrhea, are nonspecific.^5^

A few conditions may predispose an individual for otomycosis (table 1).^3^,^11^ An immunocompromised host is more susceptible to otomycosis. Patients suffering from diabetes, lymphoma, transplantation patients, patient receiving chemotherapy or radiation therapy and AIDS patients, are also at increased risk for potential complications from otomycosis^9^.

Ear cleaning habits may also contribute to pathogenesis. Traumatized external ear canal skin can present a favorable condition for fungal growth.^11^ Clinical studies have shown that otological procedures, particularly those that result in mastoid cavity, as a potential risk factor for otomycosis.^3^ The factors that predisposes to otomycosis in the previously operated ears are;

1. Recurrent drainage, antibiotic/antifungal applications – this may alter the local environment of the external ear canal and allow super infection by nasocomial fungi.
2. Alteration in the anatomy by canal wall down procedures may also produce changes in the cerumen production or relative humidity that favor fungal growth.^3^

Among the fungi, the species of Aspergillus are considered the predominant organisms implicated in the etiology of otomycosis in tropical countries.^4^ Many studies have shown that Aspergillus species was the commonly isolated fungus and Candida was next commonly isolated fungi, in immunocompetent hosts.^8^,^9^,^10^,^11^ Studies by Kishore et al^12^ and Viswanatha^13^ have shown that Candida species was mainly responsible for otomycosis in immunocompromised hosts. Infection with Candida can be more difficult to detect clinically because of its lack of characteristic appearance like Aspergillus and can present as otorrhea not responding to aural antimicrobials. Otomycosis attributed to Candida is often identified by cultural data.^3^

Pseudallescheria boydii is a saprophytic fungus capable of causing invasive fungal infections in humans, especially in immunocompetent hosts. This fungus is morphologically similar to Aspergillus but is resistant to conventional systemic antifungal therapy with amphotericin B.^14^

Many studies have shown that otomycosis is predominantly unilateral disease in immunocompetent hosts.^8^,^11^,^15^ In a recent study by Viswanatha et al^13^ bilateral involvement was seen more in immunocompromised group than in immunocompetent group.

Fungal cultures are essential to confirm the diagnosis. Hematological investigations play a very important role in confirming the diagnosis and immunity status of the patients. In diabetic patients with otomycosis, blood sugar levels should be controlled with medical therapy to prevent complications due to otomycosis. All the relevant hematological investigations should be done in immunocompromised patients.

Complications of otomycosis includes tympanic membrane perforation, hearing loss and invasive temporal bone infection (table 2).^5^,^16^ Tympanic membrane perforation can occur as a complication of otomycosis that starts in an ear with an intact ear drum.^5^ In a clinical study by Ram Kumar,^16^ the incidence of tympanic membrane perforation in otomycosis was found to be 11% and perforation was more common with otomycosis caused by Candida albicans. Tympanic membrane perforation is seen more commonly in immunocompromised patients than in immunocompetent patients.^13^ This may be due to the fact that majority of the cases of otomycosis in immunocompromised patients are caused by Candida albicans.^13^

Most of the perforations were behind the handle of the malleus. The mechanism of perforation has been attributed to mycotic thrombosis of the tympanic membrane blood vessels resulting in avascular necrosis of tympanic membrane.^5^,^17^ There may not be any clinical features predictive of tympanic membrane perforation. Tympanic membrane involvement is likely a consequence of fungal inoculation in the most medial aspect of the external canal or direct extension of the disease from adjacent skin.^3^

In immunocompromised patients malignant otitis externa can, rarely, present as an aggressive angioinvasive fungal infection of the temporal bone.^2^ Invasive Aspergillus otomastoiditis resulting from a tympanogenic source is a rare entity but is being found with increased frequency in immunocompromised hosts. Invasive Aspergillosis is most commonly observed in patients with lymphoproliferative disorders, but it may occur in variety of diseases characterized by defective humoral or cell-mediated immunity.^18^ Aspergillus and Mucor, have a propensity to invade arterial walls in immunocompromised patients, especially uncontrolled diabetes mellitus, leading to thrombosis and tissue infarction.^19^

Strauss and Fine^20^ reported two cases of Aspergillus otomastoiditis caused by Aspergillus fumigatus in AIDS patients. Haruna et al^19^ reported a case of invasive fungal temporal bone infection caused by Mucor, leading to meningoencephalitis in an immunocompromised patient. Nicolas et al^14^ have reported a case of invasive Pseudallescheria boydii fungal infection of the temporal bone in a patent with AIDS.

Treatment of otomycosis includes microscopic suction clearance of fungal mass, discontinuation of topical antibiotics and treatment with antifungal ear drops for three weeks. Ear should be kept dry for three weeks. Small perforations heal spontaneously and larger perforation requires myringoplasty.^13^

Bassouni et al^21^ studied the effects of antifungal agents and found that clotrimazole ear drops was more effective antifungal agent in the treatment of otomycosis. According to Stern et al^22^ and Jackman et al,^23^ clotrimazole ear drops is the most effective antifungal agent. In another study fluconozole ear drops was found to be more effective in treating otomycosis.^24^ Clotrimazole and fluconozole ear drops should be used for 3–4 weeks.

Oral and intravenous preparations of antifungal agents are available for severe infections in immunocompromised patients.^2^ Oral antifungal are unlikely to succeed in the absence of adequate local care.^3^ These antifungal agents should be given for 3–4 weeks depending on the patient’s condition.

Fungal infections of the mastoid cavity of the immunocompromised patients who have undergone canal wall down mastoidectomy are seen quite frequently and they require prolonged treatment with antifungal ear drops and oral antifungal drugs. The recommended treatment for patients suffering from fungal otomastoiditis includes surgical debridement and systemic antifungal therapy with amphotericin B being the gold standard.^14^ Antifungal agents should be given for 3–4 weeks depending on the patient’s condition.

## Conclusion

Diagnosis and management of otomycosis in can be really challenging in immunocompromised patients. Recurrences are common in immunocompromised patients than in immunocompetent patients. Eradication the disease may be difficult in immunocompromised patients, who have undergone canal wall down mastoidectomy. In these patient prolonged antifungal therapy is required.

Otologist should remain alert for otomycosis and should consider obtaining hematological investigations and fungal cultures when this disease is suspected in immunocompromised host.

## Figures and Tables

**Table 1:** Showing predisposing factors for otomycosis

1	Trauma
2	Relative high percentage humidity in external ear canal
3	Epithelial debris in various stages of chemical breakdown
4	High temperature which closely approximate body temperature
5	General diseases such as diabetes mellitus
6	Immunocompromised hosts
7	Increased use of topical antibiotic/steroid preparations

**Table 2:** Showing complications of otomycosis

1	Tympanic membrane perforation
2	Hearing loss
3	Invasive temporal bone infection
4	Fungal otomastoiditis
5	Meningoencephalitis
